# Bilateral Endophthalmitis in a Postpartum Immunocompetent Patient

**DOI:** 10.1155/2024/8746755

**Published:** 2024-04-24

**Authors:** Rola N. Hamam, Giulia Firmani

**Affiliations:** ^1^Department of Ophthalmology, American University of Beirut (AUB) Medical Center, P.O. Box 11-0236/D41, Riad El Solh, Beirut 11072020, Lebanon; ^2^Department of Sense Organs, Sapienza University of Rome, Policlinico Umberto I University Hospital, Via Giovanni Maria Lancisi 2, 00161 Rome, Italy

## Abstract

**Purpose:**

The aim of this report is to present the onset of bilateral endophthalmitis negative to culture testing and vitreous tapping in a postpartum immunocompetent patient.

**Methods:**

A 33-year-old patient developed floaters and severe blurry vision in both eyes 3 weeks after childbirth. With no previous surgery, no comorbidities in her clinical history, and negative diagnostic reports, endogenous endophthalmitis was suspected. Two days later, a pars plana vitrectomy was performed in both eyes one week apart, and intravitreal antibiotics and antifungals were administered during the surgery. No infectious source was identified since the cultures from the aqueous and vitreous humor returned negative in both cases.

**Results:**

Residual fibrosis around the fovea in the right eye and on the optic disc in the left eye was described. Nevertheless, the best corrected visual acuity of the patient was 20/20 in both eyes 4 months after the onset of the presumed endogenous endophthalmitis.

**Conclusion:**

This is the first report presenting a bilateral case of postpartum endophthalmitis negative to culture testing and vitreous tapping in a healthy patient with no previous surgeries nor long-term treatment. Early pars plana vitrectomy was fundamental for the correct management of this condition.

## 1. Introduction

Endogenous endophthalmitis (EE) constitutes 2-8% of all cases of endophthalmitis [1]. The causative agents of EE can be fungal or bacterial, especially fungal microorganisms which have been identified as the main culprits. The most common fungal agent causing EE is Candida. Bacteria need to be considered as well, including both Gram positive (staphylococci, streptococci) and Gram negative (Klebsiella and Escherichia coli). There are several risk factors for the onset of this condition: immunosuppression, chronic diseases such as diabetes mellitus, prolonged use of corticosteroids, underlying malignant tumors, and infections [2]. Postpartum endophthalmitis is an extremely rare condition, although other reports have mentioned this obstetric complication, but in those cases, microorganisms were still identified. We report a case of a healthy 33-year-old woman who presumably developed an EE negative to culture testing and vitreous tapping 3 weeks after a normal vaginal delivery.

## 2. Case Report

A 33-year-old healthy Middle Eastern female had an uneventful vaginal delivery in September. Systemic examination prior to delivery was unremarkable, and no risk factors were registered. On postpartum day 3, she developed mastitis which was treated by administering a dopaminergic receptor agonist (cabergoline 1 mg). A few days later, she developed fever for which she received oral antibiotic therapy (cefixime 400 mg) with no clinical improvement. By the end of the month (postpartum day 15), she underwent laboratory work-up including a complete blood count, urine exam, and chest radiograph. No signs of infection were detected despite an increase in neutrophils percentage and C-reactive protein (CRP). Gentamycin was intramuscularly administered for empirical antibiotic coverage for 7 days. Subsequently, she developed floaters and decreased vision in both eyes. On postpartum day 36, she received laboratory investigations for toxoplasma, tuberculosis, sarcoidosis, and Behçet's disease. Toxoplasma IgG were elevated whereas IgM were negative. A chest radiograph was also requested although the report was normal. Angiotensin-converting enzyme was normal, and HLA-B27 was negative while HLA-B5 was positive and ANA immunofluorescence screen assay returned with a very elevated titer (1 : 400).

**Figure 1:**
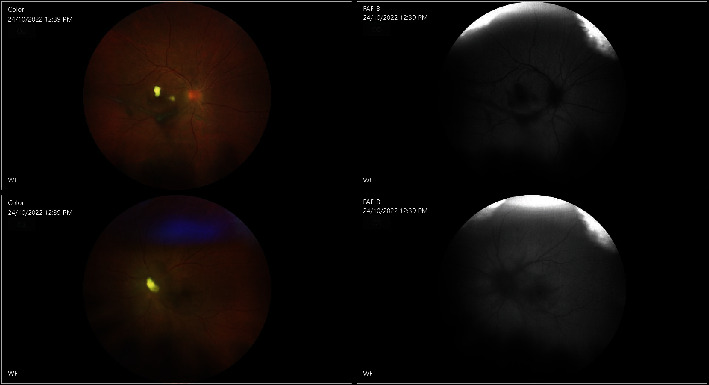
Preoperative fundus images in OD and OS.

She was referred to our department on postpartum day 42. The patient came to our attention after all systemic symptomatology has subsided. She had a visual acuity (VA) of 20/150 (+2) and 20/70, respectively, in the right (OD) and in the left eye (OS) upon presentation. Intraocular pressure (IOP) was low in both eyes. Slit-lamp examination showed injection in OS and keratic precipitates in both eyes (OU). Inflammation of the anterior chamber (AC) was also present in OU. Specifically, 1+ cells and trace flare were detected in OD while 2+ cells and 1+ flares was found in OS. Posterior synechiae were observed only in OS. Inflammation in the anterior vitreous (vitritis) was noticed as well with cells and haze. Fundus examination highlighted exudates on the fovea in OD and on the optic nerve in OS (Figure 1).

**Figure 2:**
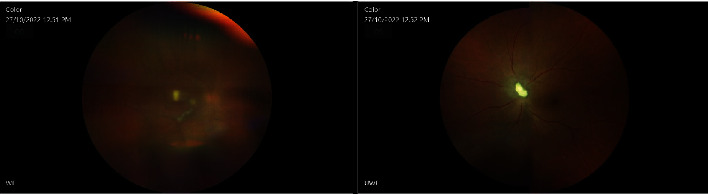
Fundus images in OD before pars plana vitrectomy and fundus images in OS after pars plana vitrectomy.

**Figure 3:**
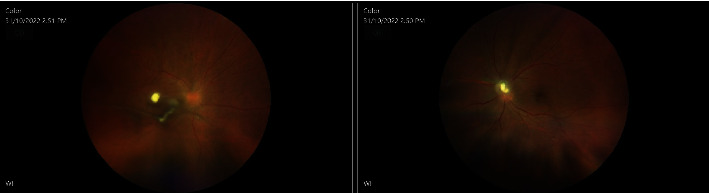
Repeat fundus images four days later in OD before pars plana vitrectomy and fundus images in OS after pars plana vitrectomy.

**Figure 4:**
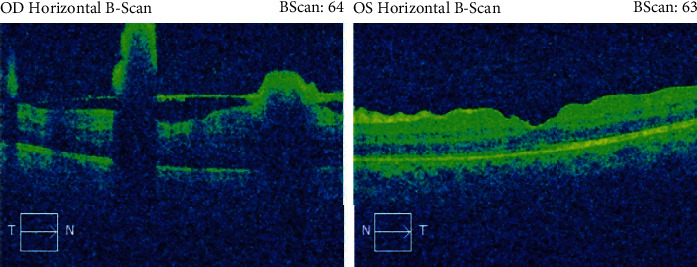
OCT in OD before pars plana vitrectomy and in OS after pars plana vitrectomy.

On postpartum day 44, she underwent pars plana vitrectomy in OS. Intravitreal antibiotic and antimycotic treatment (1 mg vancomycin, 2.25 mg ceftazidime, and 10 *μ*g amphotericin B) were administered in both eyes during surgery, and cultures were performed on the vitreous sample, in OS. Postoperative exam showed a flat retina with a swollen disc, and overlying inflammatory material was better. Optical coherence tomography (OCT) displayed a patchy ellipsoid zone loss nasally. Prednisolone acetate 1% (every 2 hours) and ofloxacin 0.3% (4 times a day) drops were given to the patient, OS. Two days after surgery (postpartum day 46), VA in OS improved to 20/50 (+2). IOP was 14 mmHg in OU. Conversely, the eye exam in OD worsened with VA reaching 20/250. Furthermore, slit-lamp examination uncovered subconjunctival hemorrhage in OD as well as a 1+ cell and hypopyon <1 mm in OD. Vitritis in OD did not show any improvement and had a string of pearls appearance. Slit-lamp examination in OS showed a 1+ cell in AC and cells and haze in the anterior vitreous. The optic disc in OS was still swollen with overlying inflammatory material (Figure 2). On that day, she received 1 mg of vancomycin and 2.25 mg ceftazidime and amphotericin B 10 microg in OU. Five days after surgery, improvement was noticed in OS (Figures 3 and 4).

**Figure 5:**
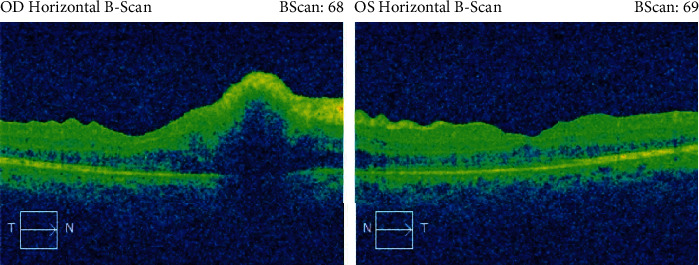
Repeat OCT seven days after the second surgery, after pars plana vitrectomy in both eyes.

**Figure 6:**
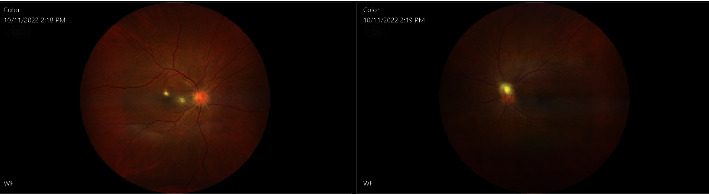
Fundus images ten days after the second surgery, after pars plana vitrectomy in both eyes.

One week later (postpartum day 51), the patient underwent pars plana vitrectomy in the other eye as well. Concurrently, intravitreal amphotericin B and moxifloxacin 0.5% were administered in OU. Samples were sent for PCR 18S and 16 RNA. The day after second surgery, the same topical therapy was given and VA improved to 20/80 (-2) in OD as well. Trace cells in AC were described during the slit-lamp examination, and the hypopyon had resolved. No vitreous clumps were observed, and retinal lesions showed resolution (Figures 5 and 6).

Five days after the second surgery (postpartum day 56), VA was 20/70 and 20/40 (-1) in OU, respectively. PCR, eye cultures (for fungal, anaerobic microorganisms), and Gram staining all returned negative. Inflammation taper drops were tapered weekly. On postpartum day 105, she was started on topical antiglaucoma drops consisting of timolol in OU BID because of a slight increase in her IOP (OD 23 mmHg, OS 26 mmHg) despite an improvement in retinal nerve fiber layer (RNFL) thickness.

**Figure 7:**
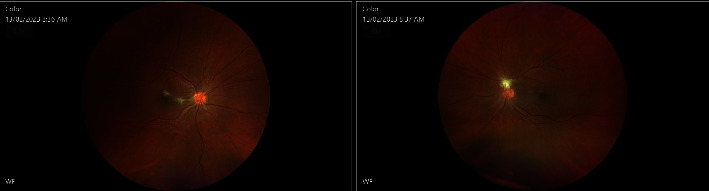
Fundus images 3 months after bilateral pars plana vitrectomy in both eyes.

**Figure 8:**
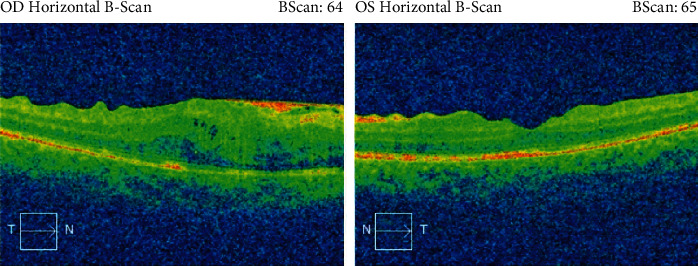
OCT 3 months after bilateral pars plana vitrectomy in both eyes.

**Figure 9:**
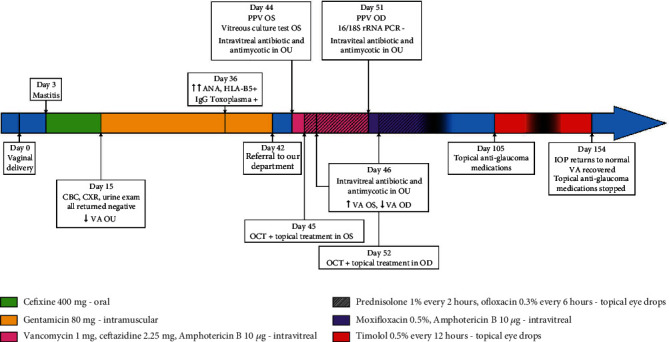
Timeline summarizing the patient's case presentation, treatments, and follow-up. Used acronyms: OU = both eyes; OS = left eye; OD = right eye; CBC = complete blood count; CXR = chest X-ray; VA = visual acuity; PPV = pars plana vitrectomy; ANA = antinuclear antibody; HLA = human leukocyte antigen; OCT = optical coherence tomography; PCR = polymerase chain reaction; IOP = intraocular pressure.

Sixteen weeks after the first ocular surgery, the patient completely recovered with VA 20/20 in both eyes. IOP remains within normal range, and treatment with IOP drops was discontinued. No inflammation was noted on the slit-lamp examination while fundus examination showed some fibrosis sparing the center of the fovea in OD as well as overlying fibrosis on the optic disc of the OS (Figures 7 and 8). A summary of the patient's case presentation and treatments is presented in the form of a timeline (Figure 9).

## 3. Discussion

The case we reported features bilateral endophthalmitis with a negative culture which occurred in a previously healthy young woman with no risk factors and no history of systemic diseases, making this scenario extremely rare. Only one case of bilateral postpartum endogenous endophthalmitis has been previously mentioned in the literature to date. Tsai et al. have described such a case in a 33-year-old woman postpartum but with a positive blood culture for Candida albicans. Moreover, systemic examination displayed several issues such as cardiomegaly acute renal failure, candidemia, and a previous Candida vaginitis [3]. Our patient had received a systemic course of antibiotics twice prior to presentation. Virologic testing for herpetic pathogens such as Varicella zoster or Herpes simplex was not performed in this patient, as common viral causes for endogenous endophthalmitis were ruled out due to inconsistency with our clinical presentation [4]. Additionally, the features of glial tissue near the optic nerve can be seen in Toxoplasma optic neuritis [5]. This is relevant, because PCR testing for toxoplasmosis from the anterior chamber was not sought. And while bilateral Toxoplasma chorioretinitis is rare, this can occur in immune or relative immune compromised individuals. MRI of the brain and orbits could have been performed to assess for optic nerve enhancement as this may have helped to confirm the diagnosis. However, because of the string of pearl appearance, we were more inclined to believe in a fungal origin of the case. Furthermore, there is evidence to suggest that positivity for HLA-B5 testing may contribute to diagnosis of the Behçet disease in some cases [6]. These, combined with Middle Eastern and the fact that pregnancy may induce flare ups, are the reasons why the Behçet disease should have been taken into consideration as a differential diagnosis [7]. However, it was ruled out based on clinical presentation. In fact, our patient did not have any oral or genital ulcers, which are accepted as “sine qua non” among diagnostic criteria [8]. Furthermore, it must be mentioned how HLA-B51 remains the most important genetic factor in the Behçet disease [9] and not HLA-B5. However, HLA-B51 testing was not deemed necessary after suspicion of the Behçet disease was excluded. For these reasons, our patient did not receive any systemic steroids. Finally, it should be mentioned how the patient was started on systemic antibiotics without blood cultures. Thus, a causative organism might have been missed. However, the negativity of PCR, eye cultures, and Gram staining and the response to the treatments she received support the possibility of endogenous endophthalmitis. Finally, the presentation of the present case may be described as “panuveitis”; however, we chose the term “endophthalmitis” given our impression that there was an infectious process as suggested by the history of fever and systemic antibiotics and response to antimicrobial treatment without the use of any steroids. Also, while it is true that endophthalmitis may present with a hypopyon up to 85% of the times, especially when acute post an intervention, up to 15% of cases present without a hypopyon [10]. To our knowledge, our case report may be the first of its kind as no other cases feature bilateralism of the endophthalmitis, linked with the postpartum condition and with no systemic comorbidities.

The other aim of our article is also to highlight the importance of undergoing early diagnostic and therapeutic PPV in the case of severe eye inflammation. Indeed, our patient has undergone this surgery two days after being referred to our department. Other ophthalmologists have chosen the same treatment option along with medical treatment [3, 9, 11–15], For instance, a diagnostic core vitrectomy was performed in a postpartum fungal endogenous endophthalmitis in a young healthy woman [16]. In addition, other authors as well have already highlighted the benefit of this surgery [17].

To the best of our knowledge, most cases related to pregnancy in literature are caused by Candida, a fungal microorganism [8, 9, 12–16, 18]. Only two cases were caused by bacteria: one by a Gram-negative (Sphingomonas paucimobilis) [19] and one by a Gram-positive microorganism (Staphylococcus aureus) [20] (Table 1). In the case we presented, all samples and cultures returned negative. In agreement with our presentation, 7 patients described by other authors also did not have any previous risk factors [11–13, 16, 18–20], whereas two had diabetes mellitus or other systemic issues [3, 15]. Three patients had a vaginal infection during pregnancy [3, 9, 16]. Our patient had a mastitis following pregnancy similarly to another case described in literature [20] and achieved a 20/20 vision during the follow-up, in agreement with other patients who obtained an overall favorable visual prognosis [11–13, 15, 16, 18, 19]. Risk factors for the development of a mastitis include nipple excoriation, maternal stress, malnutrition or chronic medical conditions, nipple piercings, smoking, a tight bra, or an improper technique while breastfeeding. However, these conditions do not apply to our patient [21, 22]. The main infectious cause of mastitis is Staphylococcus aureus [23]. However the negative result of our cultures might be related to the fact that our patient had taken antibiotics prior to presentation in our department. Ultimately, we may not be certain of the etiological origin of EE which occurred in our patient, as bacterial or fungal causes could not be confirmed through our testing, despite a strong suspicion of an underlying bacterial cause due to the history of mastitis.

## 4. Conclusion

Bilateral postpartum endogenous endophthalmitis in a healthy individual is a rare entity. A peripartum infection such as mastitis combined with severe blurry vision and ocular inflammation should raise awareness and prompt treatment. Surgical treatment must be held into consideration in those circumstances.

## Figures and Tables

**Table 1:** Review of case reports featuring postpartum endophthalmitis.

Study	Microorganism	Risk factor(s) (RF)	Eye(s) involved	Treatment	Final visual outcome
Canthrill case n°1 (1980), Ref. [14]	Candida	History of Candida vaginitis	OS	Ppv, intravitreal, and systemic antifungals	20/200
Canthrill case n°2 (1980), Ref. [14]	Candida	Fever after delivery	OS	Ppv, intravitreal, and systemic antifungals	20/50
Guex-Crosier (1993), Ref. [18]	Candida	No RF	OD	Oral fluconazole	20/20
Tsai (2002), Ref. [3]	Candida	History of Candida vaginitis, preterm delivery, acute renal failure, and cardiomegaly	Bilateral	Intravenous antifungal therapy, systemic antibiotic therapy, vitrectomy, and intravitreal antimycotics	OD 6/10 OS counting fingers (CF)
Rahman (2011), Ref. [19]	Sphingomonas paucimobilis	No RF premature rupture of membranes	OD	Intravitreal antibiotics, systemic antibiotics, and steroids	6/9
Wu (2011), Ref. [11]	Candida, Cladosporium	No RF immunocompetent, but suspicion of Candida vaginosis	OS	Ppv and intravitreal antibiotics+antifungals, systemic and topical antifungals, and topical antibiotics	20/25
Lee (2012), Ref. [13]	Candida	No RF immunocompetent	OD	Ppv, systemic, and intravitreal antifungal therapy	20/20
Sahu (2013), Ref. [12]	Candida	No RF immunocompetent	OS	Ppv and intravitreal and oral antifungals and oral antibiotics	20/20
Albloushi (2019), Ref. [15]	Candida	History of diabetes mellitus, vaginal discharge during peripartum period	OD	Ppv and intravitreal antifungals	20/40
Singh (2020), Ref. [20]	Staphylococcus aureus	No RF immunocompetent, left mastitis	OS	Systemic and intravitreal antibiotics, intravitreal steroids, topical antibiotics, and steroids	20/400
Bansal (2022), Ref. [16]	Candida	No RF immunocompetent, vaginal infection during pregnancy	OD	Diagnostic core vitrectomy and intravitreal and oral antifungals and steroids	20/30

Ppv = pars plana vitrectomy; OD = right eye; OS = left eye.

## Data Availability

Data is available on request.
